# Field-adapted sampling of whole blood to determine the levels of amodiaquine and its metabolite in children with uncomplicated malaria treated with amodiaquine plus artesunate combination

**DOI:** 10.1186/1475-2875-8-52

**Published:** 2009-03-30

**Authors:** Muhammad Ntale, Celestino Obua, Jackson Mukonzo, Margarita Mahindi, Lars L Gustafsson, Olof Beck, Jasper W Ogwal-Okeng

**Affiliations:** 1 Department of Pharmacology and Therapeutics, Makerere University, 7062 Kampala, Uganda; 2 Unit of Tropical Pharmacology, Division of Clinical Pharmacology, Department of Medical Laboratory Medicine, Karolinska University Hospital, Huddinge, Stockholm, Sweden

## Abstract

**Background:**

Artemisinin combination therapy (ACT) has been widely adopted as first-line treatment for uncomplicated falciparum malaria. In Uganda, amodiaquine plus artesunate (AQ+AS), is the alternative first-line regimen to Coartem^® ^(artemether + lumefantrine) for the treatment of uncomplicated falciparum malaria. Currently, there are few field-adapted analytical techniques for monitoring amodiaquine utilization in patients. This study evaluates the field applicability of a new method to determine amodiaquine and its metabolite concentrations in whole blood dried on filter paper.

**Methods:**

Twelve patients aged between 1.5 to 8 years with uncomplicated malaria received three standard oral doses of AQ+AS. Filter paper blood samples were collected before drug intake and at six different time points over 28 days period. A new field-adapted sampling procedure and liquid chromatographic method was used for quantitative determination of amodiaquine and its metabolite in whole blood.

**Results:**

The sampling procedure was successively applied in the field. Amodiaquine could be quantified for at least three days and the metabolite up to 28 days. All parasites in all the 12 patients cleared within the first three days of treatment and no adverse drug effects were observed.

**Conclusion:**

The methodology is suitable for field studies. The possibility to determine the concentration of the active metabolite of amodiaquine up to 28 days suggested that the method is sensitive enough to monitor amodiaquine utilization in patients. Amodiaquine plus artesunate seems effective for treatment of falciparum malaria.

## Background

Artemisinin combination therapy (ACT) has been widely adopted as first-line treatment for uncomplicated falciparum malaria [1-3]. Although these drug combinations appear to be safe and well-tolerated, experience with their use in Africa is still limited [4,5]. Uganda recently adopted ACT, whereby artemether + lumefantrine (AL, Coartem^®^) was recommended as first-line regimen for treatment of falciparum malaria. AL, however, has several limitations including twice-daily dosing regimen and recommendation to administer the drug together with fatty food [6].

Amodiaquine (AQ) is a 4-aminoquinoline drug that has demonstrated sustainable efficacy compared to other anti-malarial drugs [7-9]. In view of low cost and high efficacy, AQ has been considered as a viable anti-malarial agent despite reported low risk of agranulocytosis during long term use [10-13]. Amodiaquine plus artesunate (AQ+AS), is recommended as an alternative first-line regimen to AL for the treatment of uncomplicated malaria in Uganda and many other African countries [14]. Since AQ+AS combination is more affordable than AL, it will most likely find extensive use in the general population.

Generally, most of the malaria endemic areas are rural. These areas usually suffer from shortage of resources, for example electricity, which is required during collection of samples such as plasma. Furthermore, adsorption and drying of whole blood samples on filter paper is known to reduce the risk of exposure to HIV, hepatitis B and C virus, and other infectious agents [15], thus making sample collection and subsequent handling relatively safe.

Thus, in order perform clinical studies necessary for evaluation of AQ+AS impact in such areas, field-adapted sample collection and robust analytical techniques for monitoring AQ levels in blood and other body fluids are needed. Methods for determination of amodiaquine and its metabolite, desethylamodiaquine (DAQ) from plasma, whole blood and urine have been reported [16-18], but until recently there were none, where amodiaquine was dried on filter paper under field conditions, all previous attempts to store AQ on filter paper were futile [16,17]. The other two filter paper methods reported for AQ [19,20] were not suitable for field conditions and were expensive for resource-limited settings.

A new field-adapted filter paper method was recently developed for determination of amodiaquine from whole blood spotted on filter paper [21]. The aims of this study were to explore the practical applicability of the developed analytical procedure for determination of amodiaquine from whole blood dried on filter paper in the field, and to verify whether the measured drug levels correlate to the parasite clearance in children on treatment with AQ+AS combination.

## Methods

### Study area

The study was conducted in Aduku Health Centre, Apac District in Northern Uganda between January and February, 2008. Aduku is one of the sentinel sites that were chosen by Uganda Malaria Surveillance Project for evaluating the efficacy of available anti-malarial therapies and monitoring adverse drug events. It is surrounded by a large swamp and has very high malaria transmission intensity. The location is rural and, therefore, possibly has no drug pressure as there has been no supply of AQ or AS. The health centre receives free supplies of Coartem^® ^from government for malaria treatment.

### Recruitment of patients

Consent forms were administered to all parents/guardians whose children presented with fever to the study site during study period. Patients that consented/assented to participate in the study underwent both clinical and laboratory examination for malaria. Those suffering from malaria were further screened to exclude cases of complicated malaria (those reported to have had repeated convulsions, unable to feed orally, exhibiting central nervous system symptoms).

Twelve patients aged between 1.5 to 8 years, having clinically-confirmed acute uncomplicated falciparum malaria, were recruited into the study and appropriate referrals were made for the rest. Study participants were followed-up for 28 days according to World Health Organization (WHO) protocol [22]. Since this was not a primary efficacy study, no minimum parasitaemia was set for the inclusion criteria.

### Ethical approval

Ethical approval for this study was given by the Makerere University Research and Ethics Committee and permission to conduct the study was given by Uganda National Council of Science and Technology.

### Treatment and follow-up procedures

At enrolment, a medical history was taken and a clinical examination was made. Two venous blood samples were obtained. One sample was used to obtain solid and consistent readings of the parasites and the other for day zero drug levels. The children were thereafter orally given AQ (Amobin^®^) (Regal Pharmaceuticals Ltd., Nairobi, Kenya) plus AS (Arinate^®^) (ERFA n.v./s.a, Brussels) under supervision. Both Amobin^® ^and Arinate^® ^are made according to Good Manufacturing Practice (GMP). The medication dosages were based on the age group as recommended by the Ministry of Health Uganda [23]. All patients received standard single daily oral doses of AQ+AS for three days. Patients aged between one to three years received AQ+AS (150 mg, 50 mg), while those between four to six years received AQ+AS (200 mg, 75 mg), and those between six to 12 years received AQ+AS (300 mg, 100 mg) respectively, on each day.

Patients were asked to return to the Health Centre for follow-up on days 1, 2, 3, 7, 14, 21, 28, and on any other unscheduled day that they felt sick. Those who did not return for the scheduled follow-ups were visited at home on those days. On each visit parasitaemia was determined from thick blood smears, while 100 μL of phosphoric acid treated blood was spotted on filter paper for drug level analysis as described [21]. The thick blood smears were stained using 2% Giemsa and quantified against 200 leukocytes and parasite densities were recorded as the number of parasites/200 WBCs.

### Sample collection, preparation and analysis

On the day of recruitment (day 0), about 200 to 300 μL of venous blood samples was collected before treatment and samples were collected daily thereafter, just before administration of AQ+AS. Aliquots of collected blood were mixed with equal amounts of phosphoric acid (10%, v/v) from which 100 μL was spotted onto filter paper and allowed to dry at room temperature (23 – 28°C). The filter paper samples were then stored in plastic envelopes till analysis. The analysis was carried out as described by Ntale *et al *[21].

Thus, filter paper containing dried blood spots was cut into 4 to 6 small pieces and put into 12 mL polypropylene test tubes. Subsequently, 150 μL of I.S. solution (0.6 μM) and 1 mL of water were added and the contents were sonicated for 15 min. Sodium carbonate buffer (2 mL, 0.2 M, pH 9.7), KOH (120 μL, 1 M) and 8 mL of di-isopropylether were added. Final pH of the water phase was between 9.17 – 9.24. The samples were shaken for 20 min. and centrifuged for 10 min. at 3500 × g. The organic (upper) phase was transferred to a new polypropylene test tube and back-extracted for 10 min. with 150 μL of sodium dihydrogen phosphate buffer (0.1 M, pH 4). The phases were separated by centrifugation for 10 min. and the organic phase was removed by aspiration. The water phase (130 μL) was injected into the chromatograph.

## Results

A total of twelve children participated in the study, with age ranging between 1.5 to 8 years. There was equal sex distribution but their weight varied between 7.7 Kg to 25.8 Kg. The AQ dosing was based on age, however, on a mg/Kg basis the dose ranged from 10.1 to 19.5 (Table 1).

**Table 1:** Characteristics, parasitaemia and AQ daily dose for the 12 children with uncomplicated *Plasmodium falciparum *malaria

	Subject											
	
Characteristics	A	B	C	D	E	F	G	H	I	J	K	L
Age (yrs)	1.5	1.5	1.7	2.3	2.5	2.5	3	3	3	4	8	8
Sex	M	F	M	F	F	F	F	M	M	M	F	M
Weight in Kg	10.2	7.7	11.2	13	11.8	12	13.2	11.7	14.9	16.1	25.8	20.2
AQ Dose (mg/Kg)	14.7	19.5	13.4	11.5	12.5	10.1	12.7	12.8	11.4	12.4	11.6	14.9
												
Parasitaemia (/200 WBCs)												
Day 0	600	7,000	7,500	650	9,000	741	13,000	460	8,000	9,471	400	1,000
Day 1	30	800	1,720	200	721	47	600	4	129	437	7	82
Day 2	0	0	0	0	0	0	0	0	0	0	0	2
Day 3	0	0	0	0	0	0	0	0	0	0	0	0
Day 7	0	0	0	0	0	0	0	0	0	0	0	0
Day 14	0	0	0	0	0	0	0	0	0	0	0	0
Day 21	0	0	0	0	0	0	0	0	0	0	0	0
Day 28	0	0	0	0	0	0	0	0	0	0	0	0

### AQ and DAQ concentrations

**Figure 1:**
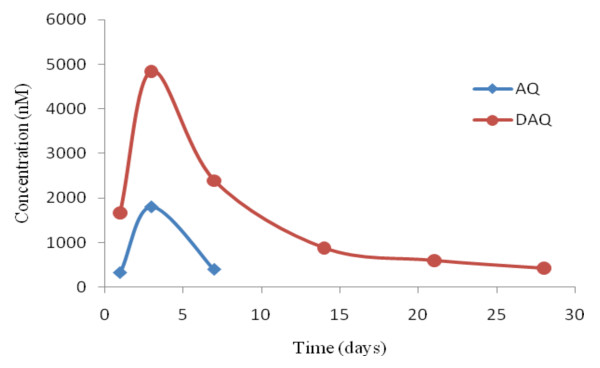
**Median concentration-time profile for AQ and DAQ of 12 children on treatment with AQ+AS combination**.

In all subjects no AQ or DAQ was detected in the pre-dose blood sample. The AQ level could be measured by day 3 in all subjects, but by day 7 only eight subjects had quantifiable levels. DAQ was quantifiable by day 28 in all subjects with a median concentration of 227 nM (68–1282). Median AQ levels were highest on day 3, and DAQ levels followed a similar trend but were higher than those of AQ at all time points (Table 2 and Figure 1). The lowest observed concentration for AQ was 178 nM and this was on day 7, and for DAQ, it was 68 nM observed on day 28, while the highest concentrations of AQ (5,312 nM) and DAQ (12,018 nM) were observed on day 3.

**Table 2:** Whole blood concentrations (median values and range) of AQ and DAQ for children (n = 12) at different times before and after administration of AQ+AS

	Time (days)						
	
Analyte	d0	d1	d3	d7	d14	d21	d28
AQ (nmolL^-1^)	0	321 (115–1696)	1810 (1736–4087)	395 (68–854)	0	0	0
DAQ (nmolL^-1^)	0	1675 (422–6098)	4850 (1755–12018)	2395 (551–5423)	882 (402–2523)	602 (395–1448)	227 (68–1282)

## Discussion

Due to renewed interest in AQ as a therapeutic agent, there is a need for a sensitive field-adapted and affordable analytical method for its determination to enable clinical pharmacokinetic studies and monitoring of exposure to AQ in a specific population. Pilot results from an earlier developed method showed that it was possible to determine AQ and its main metabolite DAQ concentrations in three healthy subjects up to at least 24 hours after drug intake. This study aimed to assess field applicability of the sampling procedure as well as the sensitivity of the method. In addition, the parasite clearance and achieved drug levels in Ugandan children on treatment with AQ + AS were analysed.

To control for any anti-malarial drug consumption in the target population before treatment, AQ and DAQ concentrations were measured at day zero (before medication) in 12 whole blood samples collected from all the patients. The enrolled patients were followed-up for 28 days and during follow-up, none of the subjects dropped out. The failure to quantify AQ after seven days in all the subjects confirmed that the drug was rapidly metabolized to its active metabolite, DAQ as reported earlier [24].

Although the AQ dose varied from 10.1 to 19.5, there were no adverse drug reactions observed. The observed variability in the reported concentrations of AQ and DAQ could be attributed to differences in body weight of the study participants and other factors such as nutritional status and inter-individual genetic variability. This finding is in agreement with existing literature, which also reveals considerable variability between patients and healthy subjects after intravenous infusion with AQ. Post-infusion plasma concentrations ranging between 231 to 2,352 nM for AQ have been reported in patients while values between 180 to 5,400 nM have been detected in healthy individuals [25,26]. In other studies, it has been reported that AQ was not detectable in plasma beyond day 3 post-dose [26-28]. The findings of this study show that AQ was present in whole blood seven days after drug intake, a confirmation that whole blood levels of the drug are higher than the corresponding plasma levels as earlier reported [28]. This could most probably be explained by the fact that amodiaquine, like chloroquine, also concentrates in the erythrocytes and binds to platelets and leucocytes.

Despite inter-individual variations in AQ and DAQ concentrations found in this study, the results demonstrate that both the sampling and analytical procedure of the recently developed filter paper method for determination of AQ and DAQ are applicable in the field. In addition, the analytical procedure is sufficiently sensitive for the analysis of low concentrations of both AQ and DAQ in whole blood dried on filter paper.

It has been suggested earlier that DAQ (and not AQ) must be monitored *in vivo *[26]. Therefore, the possibility to determine desethylamodiaquine up to 28 days with this method will enable future population-based studies in countries where amodiaquine in combination with artesunate have been adopted as an alternative first-line treatment to AL. The study also adds to the documentation of AQ levels in children on treatment with AQ+AS.

Although the present study was not powered enough (n = 12) to assess efficacy, it is encouraging that the 28-day follow up indicated 100% parasitological cure for the 12 patients treated with AQ+AS (table 1). This could probably be explained by the high blood levels of DAQ achieved during the course of treatment. The high levels of the metabolite may also be essential in curbing problems due to re-infections. The results demonstrate that the AQ+AS combination is still very effective in treating uncomplicated malaria attacks in children less than eight years old living in an area of Uganda to which the disease is highly endemic.

## Conclusion

This study has demonstrated that under field conditions it is possible to collect and analyse blood samples dried on filter paper thus making it possible to carry out large scale study of AQ pharmacokinetics in children with malaria infection, and to follow-up for any severe adverse reactions related to drug/metabolite concentrations in blood.

## Competing interests

The authors declare that they have no competing interests.

## Authors' contributions

MN was responsible for the conceptualization and design of the study, developing the research protocol, data collection and analysis and overall responsibility for the preparation of the manuscript. CO participated in the study design, protocol development, data collection, analysis and manuscript preparation. MM participated in experimental design, analysed all the samples and gave comments on the manuscript. JM, LLG, OB and JOO all contributed to the design of the study and to the preparation of the manuscript.
